# Solvent-free synthesis of coumarin derivatives for improved anti-corrosion and mechanical performance of primer coatings

**DOI:** 10.1038/s41598-026-55719-y

**Published:** 2026-06-16

**Authors:** Anhar Abdel-Aziem, W. A. Hussein, Mona A. Ahmed, M. M. Elsawy

**Affiliations:** 1 Department of Chemistry, Faculty of Science (Girls), Al-Azhar University, P. O. Box 11754, Nasr City, Cairo Egypt; 2 Egyptian Petroleum Research Institute (EPRI), 11727 Nasr City, Cairo Egypt

**Keywords:** Green synthesis, Coumarin derivatives, Primer coatings, Corrosion inhibition, Dynamic mechanical analysis (DMA), Thermogravimetric analysis (TGA), Quantum chemical calculations, Chemistry, Engineering, Materials science

## Abstract

In the current study, an environmentally friendly and energy-efficient synthesis of two coumarin derivatives was achieved using a solvent-free grinding technique. This green protocol offers several advantages over conventional methods, including shorter reaction times, the absence of hazardous solvents, mild conditions, straightforward workup, low cost, and high product yields. The chemical structures of the synthesized derivatives were confirmed by IR, $\:1\mathrm{H}$NMR, $\:13\mathrm{C}$NMR, and mass spectrometry. The coumarin-based corrosion inhibitors (compounds **3** and **6)** were incorporated into a commercial solvent-based primer to enhance its protective properties against corrosion. Dynamic Mechanical Analysis (DMA) revealed an increase in storage modulus (E′) and thermal resistance, particularly in the formulation modified with the pyrazole-containing compound 6, which retained stiffness at elevated temperatures. Complementary thermogravimetric analysis (TGA) confirmed improved thermal stability, as shown by higher degradation onset temperatures and greater char yields in the modified coatings. The inclusion of these compounds significantly improved the mechanical performance of the coated films. Notably, the formulation containing compound **6** exhibited superior hardness, impact resistance, and adhesion, attributed to strong interfacial interactions with the primer matrix. The anticorrosive performance of the modified primers was further evaluated using salt spray testing. Both compounds significantly enhanced corrosion resistance compared to the unmodified primer, with compound **6** providing superior protection. These findings were supported by quantum chemical calculations, which showed that compound **6** exhibited a lower energy gap (ΔE = 1.915 eV), indicating higher reactivity and stronger interaction with the metal surface. Overall, the study demonstrates the dual function of coumarin-based corrosion inhibitors in enhancing both the mechanical integrity and corrosion resistance of commercial primer coatings.

**Supplementary Information:**

The online version contains supplementary material available at 10.1038/s41598-026-55719-y.

## Introduction

Metallic corrosion represents a severe global economic challenge, with costs exceeding USD 2.5 trillion annually, accounting for approximately 3.4% of the global GDP^1^. In the oil and gas sector, carbon steel serves as a primary and economical material for pipelines and containers owing to its superior mechanical properties^2,3^. However, its susceptibility to corrosion in saline, acidic, and oxidizing environments poses severe threats to operational safety, structural integrity, and economic viability^4,5^. Corrosion inhibitors—specialized chemical additives are extensively employed to alleviate these problems^6,7^. Among the diverse strategies employed for corrosion mitigation; including cathodic protection, surface passivation, alloy modification, and coatings — organic corrosion inhibitors have attracted sustained scientific interest due to their tunability, cost-effectiveness, and capacity for film-forming protection at low concentrations^6,7^. These inhibitors function primarily by adsorbing onto the metal surface, creating a protective hydrophobic barrier that restricts active cathodic and anodic reaction sites^8–12^. The inhibition efficacy is governed by the presence of heteroatoms (O, N, S), and π-electron clouds, polar functional groups, and conjugated aromatic rings, which serve as reactive coordination centers with the metal d-orbitals^13–17^.

Coumarin (2 H-chromen-2-one) derivatives represent a privileged class of heterocyclic scaffolds with broad industrial relevance, encompassing applications in food flavoring, dyes, fragrances, pharmaceuticals, and agrochemicals^18–20^. These heterocycles possess a dense network of delocalized electrons and reactive carbonyl oxygen atoms. Recent literature indicates that incorporating supplementary heteroatoms, aryl rings, and thiosemicarbazide motifs into the coumarin framework drastically improves its electron-donating capacity^21,22^. Consequently, coumarin-based compounds can form stable, chemisorbed protective layers on ferrous and non-ferrous metal surfaces, with inhibition efficiencies often exceeding 90% in aggressive environments^23–25^. This structural optimization facilitates the formation of a highly stable, adherent protective film on the metal surface, making coumarin-based molecules exceptionally effective at blocking corrosive species in hostile media^26,27^. However, traditional functionalization of coumarins often suffers from prolonged reaction times, hazardous organic solvents, and toxic waste generation. To align this work with the principles of Green Chemistry, a solvent-free mechanochemical approach was utilized to synthesize two novel chromene derivatives.

Protective primer coatings form the primary defensive barrier against atmospheric and immersion corrosion, and their performance is critically dependent on the mechanical integrity, adhesion, and barrier properties of the coating film^28,29^. The incorporation of active corrosion inhibitors into primer matrices offers a synergistic protection strategy: the coating provides a physical barrier while the inhibitor molecules, released or adsorbed at coating defects and interfaces, provide electrochemical protection. Despite the considerable body of literature on solution-phase coumarin inhibitors, very few studies have systematically investigated the dual role of coumarin derivatives in simultaneously improving the mechanical performance (storage modulus, glass transition temperature, thermal stability) and the anti-corrosion efficacy of primer coatings.

While coumarin derivatives are well-studied as liquid-phase inhibitors, their use as solid-phase additives embedded in polymer coatings remains largely unexplored. To bridge this gap, new chromene derivatives **3** and **6** were integrated into a commercial primer to develop a dual-functional coating targeting simultaneous mechanical reinforcement and corrosion protection. The hybrid coating’s performance, interfacial adsorption, and surface morphology were systematically evaluated in 5% NaCl) using salt spray test.

To the best of our knowledge, no previous study has reported the simultaneous enhancement of thermo-mechanical performance and corrosion resistance of commercial primer coatings using solvent-free synthesized brominated coumarin derivatives validated through both experimental characterization and density functional theory (DFT) calculations. Therefore, this work introduces a sustainable and multifunctional coating strategy based on environmentally benign mechanochemical synthesis, offering enhanced coating durability, thermal stability, and anti-corrosion performance for potential applications in marine, oil and gas, and industrial protection systems.

## Experimental

### Instruments and materials

The melting points of the produced coumarins were determined on an electrothermal apparatus. The chemicals and solvents used for synthesis were sourced from Sigma-Aldrich in Missouri, USA, and were used as received. IR spectra (KBr disks) were recorded on a Shimadzu FT-IR 8201 PC spectrophotometer. ^1^H and ^13^C NMR spectra were recorded in CDCl_3_ and (CD_3_)_2_SO solutions on a Mercury-300 MHz spectrometer, and chemical shifts are expressed in δ ppm units using TMS as an internal reference. Mass spectra were recorded on a GC-MS QP1000 EX Shimadzu. Elemental analyses were carried out by the Microanalytical Research Centre, Faculty of Science, Cairo University.Dynamic mechanical analysis (DMA; Triton Technology-TTDMA, Mansfield, MA, USA), thermogravimetric analysis (TGA; NETZSCH STA 449 C instrument, New Castle, DE, USA) with a temperature rate of 10 °C/min, under dynamic flow of nitrogen 20 mL/min., The electrochemical analyses were conducted on the OrigaMaster 5 workstation. The salt spray was done in a chamber manufactured by CW Specialist Equipment Ltd., 20 Model SF/450, (London, UK)

All chemicals and solvents were purchased from Sigma-Aldrich in Missouri, USA, and didn’t require additional purification. The use of an environmentally acceptable solvent, simple and practical operation (filtering and washing without column chromatography separation), and excellent yields are some advantages of this technology. The foundation coating substance was a commercial-grade Rust-Oleum Primer Surfacer that was bought from Pachin, Egypt.

### Statistical analysis

All experimental measurements were conducted in triplicate, and the reported values represent the mean ± standard deviation. Reproducibility of the coating performance was confirmed through repeated measurements under identical experimental conditions.

### Synthesis of 2-(1-(6-bromo-2-oxo-2 H-chromen-3-yl)ethylidene)hydrazine-1-carbothioamide (3) and 3-(6-bromo-2-oxo-2 H-chromen-3-yl)-pyrazole-1-carbothioic acid hydrazide (6)

A mixture of 6-bromo-3-acetyl-chromen-2-one (**1)** (10 mmol) and thiosemicarbazide (**2**) (10 mmol) (or enaminone **4** and thiocarbohydrazide **5**, 10mmol each) were ground manually in a clean mortar with pestle at room temperature with four drops of glacial acetic acid. Grinding was continued for 10 min until a colored solid formed. The solid was washed with ethanol (20 mL), filtered, and recrystallized from 30 mL ethanol to afford the target compounds in good yield.

Reddish brown; Yield: 93%; m.p: 223–25 °C; FT-IR (KBr): 3313 broad(NH_2_), 3193 (NH), 1737 (C = O), 1605 (C = N); ^1^H NMR (DMSO-***d***_***6***_) (Fig s1) δ: 6.24 (s, NH_2_), 7.30 (d, 2 H, *J* = 8.1 Hz), 7.62–7.71 (m, 4 H), 7.73 (s, 1 H), 8.48 (s, NH); ^13^C NMR (DMSO-***d***_***6***_) (Fig. s2) δ: 116.89, 118.80, 121.19, 128.08, 131.80, 135.33, 140.74, 146.14, 153.09, 157.54, 159.07 (Ar-C), 174.42(C = O), 195.46 (C = S). MS m/z (Fig. s3) (%): 365 (15.72), 363 (9.28), 63 (100). Anal. calcd. for C_13_H_9_BrN_4_O_2_S (365.21): C, 42.75; H, 2.48; Br, 21.88; N, 15.34; S, 8.78. Found: C, 42.83; H, 2.55; Br, 21.95; N, 15.41; S, 8.69.

### Safety consideration

All reactions were performed following standard laboratory safety protocols. Hazardous reagents and volatile solvents were handled in a fume hood with appropriate personal protective equipment (gloves and safety glasses). No unexpected safety issues were observed during the syntheses.

### Scalability

The described grinding method is suitable for small-scale synthesis. For larger-scale preparation, mechanical grinding or solution-phase reactions can be applied without significant modification, maintaining comparable yields and purity.

### Coating composition and film preparation

Compounds **3** and **6** were synthesized as previously described and subsequently incorporated into the commercial solvent-based primer formulation (Rust-Oleum Primer Surfacer) as corrosion inhibitors to evaluate their effect on the overall coating performance.

The formulation consisted of 75 wt% primer and 20 wt% compound 3 or 6. White spirit (4 wt%) was added to control the viscosity and facilitate application. Additionally, a dispersing agent (1 wt%) was included to ensure homogeneous distribution of the additives within the primer matrix. The components were mixed thoroughly using a mechanical stirrer at room temperature for 30 min until a uniform and stable formulation was obtained^28,29^.

Table 1 outlines the full composition of the prepared coating formulations.

**Table 1:** Coating Formulation Using Rust-Oleum Primer Surfacer.

Component	Weight% (%)	Function
Rust-Oleum Primer Surfacer	75%	Base binder and adhesion promoter
Compound 3 or 6	20%	Corrosion inhibitor
White spirit (or mineral spirits)	4%	Solvent - viscosity adjustment
Dispersing agent	1%	Improves uniform dispersion of solids

Mild steel specimens were treated with the developed primer formulation, which contained compounds 3 or 6, according to a defined protocol^28,29^. Mild steel panels were initially cut to either 2 × 2 cm or 5 × 5 cm in size. Then, using 400–600 grit emery paper, their surfaces were physically abraded to get rid of any scale, corrosion, or prior coatings. After being abraded, the panels were thoroughly cleaned with distilled water, allowed to dry naturally, and then degreased by submerging them in acetone for ten minutes to remove organic impurities. A consistent wet film thickness of roughly 100–120 μm was then achieved by applying the primer formulation by brushing or dip-coating, resulting in a dry film thickness of 60–80 μm after curing. Care was taken to prevent air bubbles or discontinuities in the film from forming during application.

After 24 h of dust-free air drying at room temperature (25 ± 2 °C), the coated samples were accelerated-cured for one hour at 60 °C in a drying oven to guarantee full solvent evaporation and appropriate film formation. The panels’ surface homogeneity and the lack of flaws like peeling, pinholes, or cracks were examined visually after they had dried. The coated specimens were then kept in a desiccator until they could be further examined and tested.

### Painted samples characterization

#### Physical–mechanical properties of the dried coated films

- The viscoelastic properties of the prepared epoxy coatings, including the damping factor (tan δ) and storage modulus (E′), were evaluated using a dynamic mechanical analyzer (DMA; Triton Technology-TTDMA, Mansfield, MA, USA). Measurements were conducted at a frequency of 1 Hz in three-point bending mode. Rectangular specimens with dimensions of 25 mm × 10 mm × 3 mm were molded specifically for this analysis, allowing reliable assessment of the temperature-dependent viscoelastic behavior of the composite films. This specimen design ensured accurate comparison of the mechanical performance associated with each coating formulation^30^.
- The thermal stability of both the uncoated epoxy and the cured epoxy films was investigated using thermogravimetric analysis (TGA) with a TGA1-Mettler Toledo instrument. Samples were heated from 25 °C to 700 °C at a constant rate of 10 °C min⁻¹. The resulting TGA thermograms provided detailed insight into the thermal degradation behavior and overall thermal stability of the primer coatings^31,32^.
- Thermogravimetric analysis (TGA) was performed to assess the thermal stability and decomposition behavior of the neat primer and the formulations modified with coumarin derivatives 3 and 6. The TGA curves (Fig. 3) show a progressive mass loss with increasing temperature for all samples. Both modified coatings exhibited improved thermal stability compared to the unmodified primer.
- Notably, the formulation containing compound 6 displayed the highest thermal resistance. The onset degradation temperature shifted significantly to a higher value, and the char yield at 700 °C was also markedly increased. This behavior is attributed to the rigid, conjugated pyrazole–coumarin framework in compound 6, which restricts polymer chain mobility and promotes the formation of a thermally stable network. In contrast, the formulation containing compound 3 showed moderate improvement, due to polar functional groups that enhance bonding but are less thermally stable due to the flexible ethylidene bridge and labile C = S, N–N bonds. These findings are consistent with the DMA results in Sect. 3.1.1, and together confirm the reinforcing effect of compound 6 on the thermal performance of the coating system.

#### Evaluation of film characteristics

According to ASTM: D5895, coatings, or the resulting films, were dried at ambient temperature as well as at higher temperatures (each for one hour at 80 °C, one hour at 100 °C, and one hour at 120 °C). Standard procedures were followed for water, alkali resistance (10% NaOH) ASTM: D1647–89, acid resistance (10% HCl and 20% H_2_SO_4_) I.S 1950,159, and solvent resistance (acetone, ethanol, methanol, ethyl methyl ketone, and toluene) ASTM: D1647–59^33^.

The synthesized compounds were treated with the appropriate concentrations of cobalt driers and lead octoate to get a brushable consistency. To create a uniform coat, the coating was brushed onto glass panels and mild steel that had previously been prepared. The film’s properties were measured using standard methods after tack-free drying, such as impact ASTM E23, gloss ASTM D523, adhesion (tape test) ASTM D3359, scratch hardness by pencil test ASTM D3363, and bending test ASTM E290^28,29^.

#### Salt spray test

A salt spray test was conducted to evaluate the corrosion resistance of the coatings on carbon steel panels in accordance with ASTM B117-19 standards^33^. The coated panels, which had a scribe mark, were exposed to a 5 wt% NaCl solution at 37 °C for 720 h inside a CW Specialist Equipment Ltd. 20 Model SF/450 chamber. The corrosion resistance of the panels was appraised at the scribed area using ASTM D-1654^34^ to measure the degree of corrosion-related failure. After the exposure period, the samples were carefully removed and rinsed with deionized water to remove any salt deposits, then air-dried. All rust was thoroughly removed from the scribed surfaces. The surface’s corroded area is then visually compared against photographic reference standards to ascertain the percentage of corrosion. This method provides a reliable way to compare the corrosive performance of different coatings or material systems after being exposed to a corrosive environment.

#### Molecular reactivity

Using density functional theory (DFT), quantum chemical calculations were performed to optimize the complete geometry of coumarins **3** and **6**^35–37^.

## Results and discussion

**Fig. 1:**
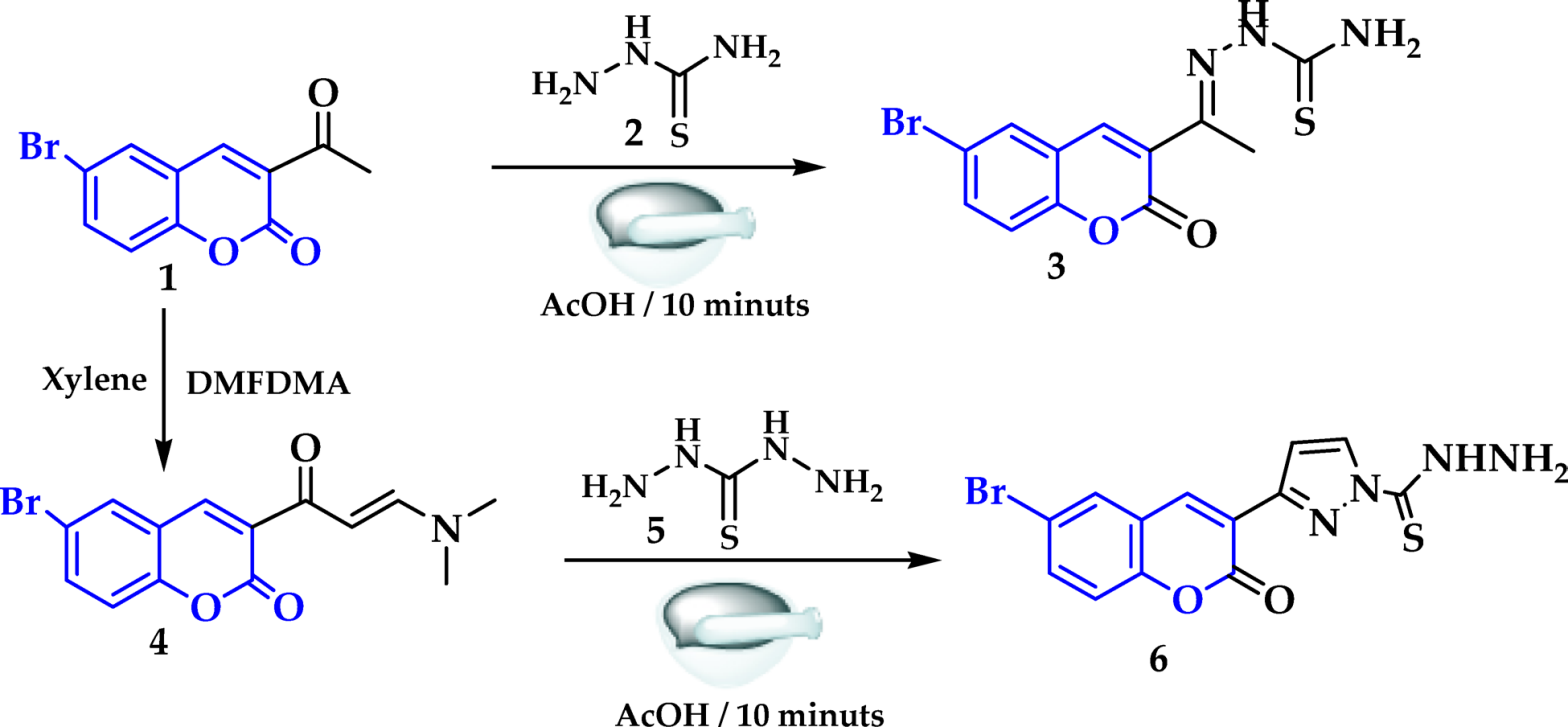
Synthesis of coumarins 3 and 6 by the grinding method.

The synthetic pathway for the preparation of target compounds is illustrated in Scheme 1. The traditional methods for the construction of coumarin derivatives suffer from low yields, long reaction time, high costs, use of harmful solvents and catalysts, large amount of energy, and release of toxic substances in the environment^38–45^. To overcome the problems of traditional methods, there is an increasing demand from chemists for developing green, efficient protocols. Recently, the grinding technique was applied for synthesis of heterocyclic compounds just by mixing the reactants manually using a porcelain mortar and pestle under solvent-free conditions^46–50^. 6-Bromo-3-acetyl-chromen-2-one **(1)** was used as a starting material in this study, as presented in Scheme 1. Earlier, hydrazine-1-carbothioamide derivative **3** was synthesized by a traditional method via stirring in ethanol. Herein, we applied a green protocol for the formation of compound **3** by manually grinding equimolar amounts of compound **1** with thiosemicarbazide (**2**) in a mortar with pestle at room temperature for 10 min in four drops AcOH. Furthermore, the target 3-(6-bromo-2-oxo-2*H*-chromen-3-yl)-pyrazole-1-carbothioic acid hydrazide (**6**) was readily accomplished through grinding a mixture of 6-bromo-3-(3-dimethylamino-acryloyl)-chromen-2-one (**4**) (which was prepared by reaction of 6-bromo-3-acetylcoumarin **1** with dimethylformaimd-dimethylacetal (DMFDMA) in dry xylene under reflux) and thiocarbohydrazide (**5**) in a mortar with pestle at room temperature for 10 min in the presence of four drops of acetic acid (Fig. 1). It was clear that the grinding method gave excellent yield (90 and 93%) and in a very short time (10 min). The spectral data of compound **6** are in agreement with the chemical structure. The ^1^H NMR in (DMSO-*d*_*6*_) (Fig. s1) registered singlet signals at δ 6.24 and 8.48ppm for NH_2_ and NH protons, respectively. The doublet and multiplet signals at δ 7.30 and 7.61ppm are accounted for by two and four aromatic protons, besides one singlet signal at δ 7.73 for CH-coumarin. In the mass spectrum of compound **6** (Fig. s3), a molecular ion peak at m/z = 365 [M + 2] and 363 [M+] is observed, which corresponds to Br^81^ and Br^79^. The predominant peak at m/z = 63 represents 100% relative abundance.

### Mechanical & chemical resistances

The primer formulation was modified to include compounds 3 and 6 to assess the efficacy of the synthesized compounds. The resulting coatings were then put through a battery of mechanical, thermal, and corrosion resistance tests.

#### Dynamic mechanical analysis (DMA) of epoxy composites with fillers 3 and 6

**Fig. 2:**
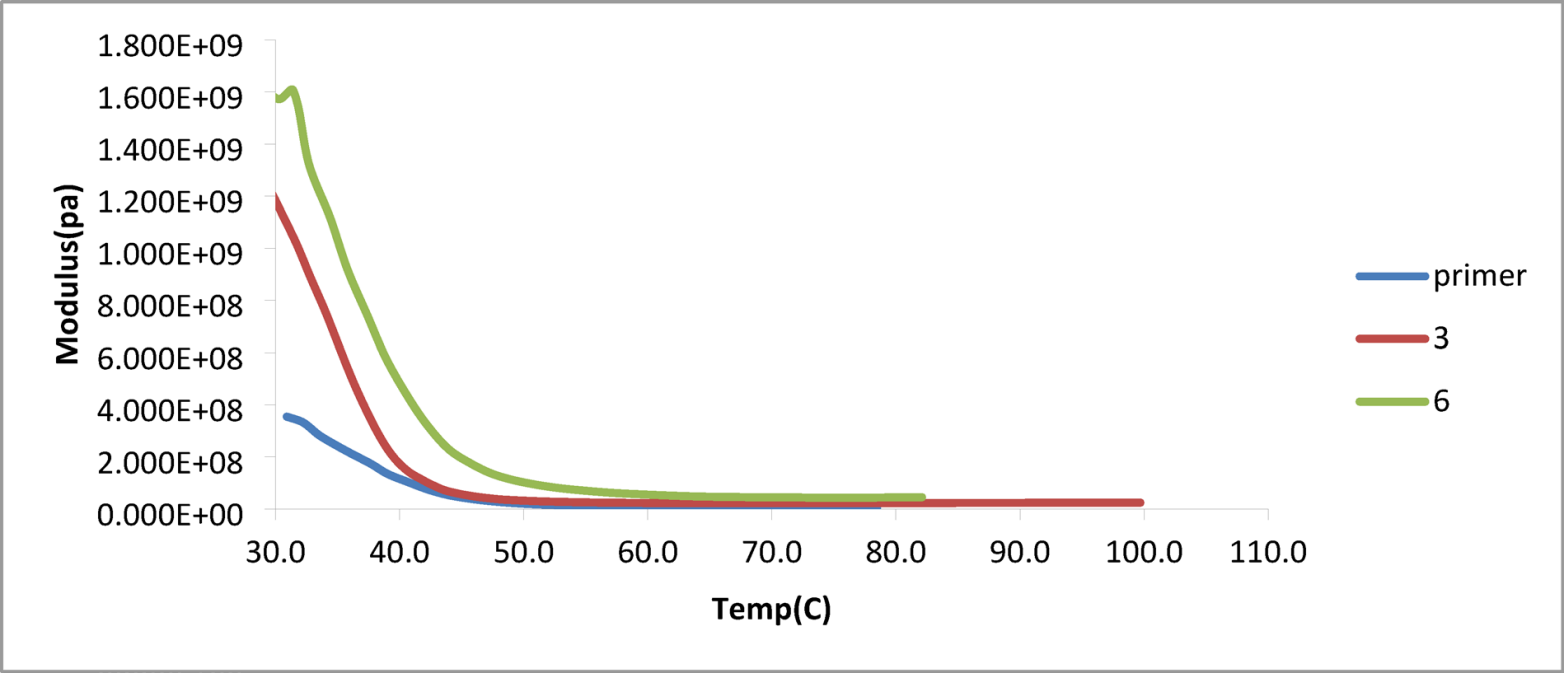
Comparative DMA curves of primer formulations with and without coumarin-based inhibitors, illustrating the superior viscoelastic performance of the composite modified with compound 6.

Dynamic Mechanical Analysis (DMA) was conducted to evaluate the influence of the two fillers on the viscoelastic behavior of the commercial primer. The storage modulus (E′)–temperature profiles, illustrated in Fig. 2, the results reveal that the incorporation of compounds 3 and 6 enhances the stiffness of the primer formulation compared to the unmodified (neat) primer. This improvement reflects more efficient stress transfer and stronger interfacial adhesion between the fillers and the epoxy matrix^51^. The DMA results indicate that both coumarin-based inhibitors (compounds 3 and 6) significantly enhance the storage modulus (E′) of the primer formulation across the entire temperature range. However, the extent of mechanical reinforcement and thermal stability varies depending on the molecular structure of each compound.

**Compound(3)** (2-(1-(6-bromo-2-oxo-2 H-chromen-3-yl)ethylidene)hydrazine-1-carbothioamide) contains flexible ethylidene linkages and polar groups such as –NH, C = O, and C = S, which can form hydrogen bonds with hydroxyl and ether groups in the epoxy network. These polar interactions enhance rigidity and stiffness at lower temperatures. Nevertheless, due to the lower degree of conjugation and structural flexibility of the ethylidene bridge, the material exhibits a more pronounced decrease in storage modulus with increasing temperature, reflecting limited thermal reinforcement^52^.

In contrast, **Compound(6)** (3-(6-bromo-2-oxo-2 H-chromen-3-yl)-pyrazole-1-carbothioic acid hydrazide) contains a rigid pyrazole ring, which provides extended aromatic conjugation and multiple interaction sites capable of forming strong hydrogen bonds and π–π stacking interactions with components of the primer matrix.

These structural features restrict polymer chain mobility, leading to improved load transfer and higher storage modules even at elevated temperatures. The slower decay of E′ with temperature indicates that the pyrazole-containing filler improves the crosslink rigidity and thermal resistance of the composite^53,54^.

Overall, both coumarin derivatives improve the mechanical and thermo-mechanical performance of the primer by enhancing interfacial adhesion and restricting molecular mobility. However, **Compound 6** exhibits superior reinforcement efficiency and thermal stability due to its rigid, conjugated heterocyclic structure and stronger molecular interactions with the primer matrix^55^. These findings suggest that the molecular rigidity and conjugation of the inhibitor structures play a key role in governing the viscoelastic performance and durability of the modified primer formulations.

####  Thermogravimetric analysis (TGA): effect of coumarin derivatives on thermal stability

**Fig. 3:**
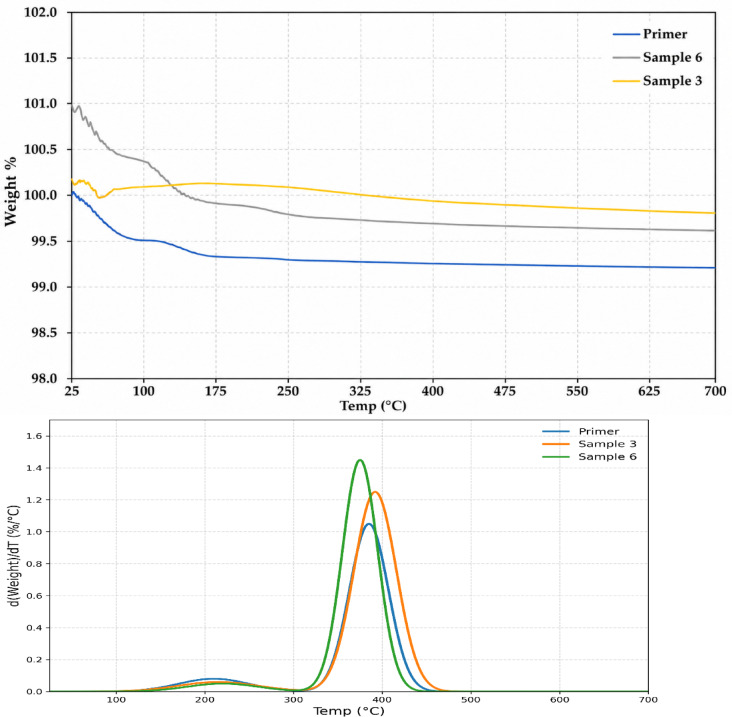
(**a**) TGA curves and (**b**) DTG curves of the neat commercial primer and primer formulations modified with coumarin derivatives (Sample 3 and Sample 6) recorded from 25 to 700 °C.

Thermogravimetric analysis (TGA) was performed to assess the thermal stability and decomposition behavior of the neat commercial primer and the modified formulations containing coumarin derivatives 3 and 6^56,57^. The TGA and DTG curves (Fig. 3) show progressive mass loss as temperature increases, while the DTG curves provide additional information regarding the degradation rate and thermal decomposition stages of the investigated formulations. Both modified composites exhibited improved thermal stability relative to the unmodified primer. This behavior is consistent with the DMA results, where compound 6 increased the storage modulus and glass transition temperature (Tg) more effectively.

The pyrazole-based compound 6 not only increases stiffness and glass transition temperature, but also raises the decomposition onset temperature and char yield^31,32^, indicating a more thermally stable and durable primer formulation compared with both the neat primer and the hydrazine-carbothioamide-based compound 3. The onset of degradation shifted toward higher temperatures for both modified primer samples, reflecting enhanced thermal stability. This improvement may be attributed to stronger intermolecular interactions between the coumarin derivatives and the primer matrix, which restrict polymer chain mobility and retard thermal decomposition^58^.

Sample **6** showed the highest thermal stability, consistent with the DMA results, where it also exhibited a higher storage modulus. This suggests that the improved stiffness and restricted molecular motion observed in DMA contribute to enhanced heat resistance in TGA analysis. Furthermore, the DTG curve of Sample 6 exhibited a lower degradation rate and a broader degradation peak compared with the neat primer, confirming its improved resistance toward thermal decomposition^59^.

Compound **6** exhibited the smallest total weight loss and the highest onset temperature of decomposition among the investigated samples, indicating the greatest thermal resistance. This improved stability is attributed to the rigid aromatic pyrazole–coumarin structure and stronger interactions between the compound and the primer matrix. The conjugated heterocyclic framework and the presence of heteroatoms (N and S) promote the formation of a thermally stable network and facilitate char formation, both of which help suppress volatile degradation.

Compound **3** showed intermediate behavior; it improved thermal stability compared with the neat primer but was less effective than compound 6. Its hydrazine-carbothioamide structure can form hydrogen bonds with the polymer matrix and enhance crosslinking at moderate temperatures; however, its flexible ethylidene bridge and labile functional groups (N–N and C = S) are more prone to thermal cleavage, leading to earlier mass loss. The neat primer decomposed at lower temperatures and exhibited the lowest residual mass, consistent with a less crosslinked and thermally less stable polymer matrix.

#### Evaluation of primer coatings modified with coumarin derivatives in terms of their mechanical and chemical properties

Table 2 provides a summary of the standard mechanical and chemical tests that were performed to further assess the coating formulations’ performance. Along with chemical resistance tests in a variety of media, including water, organic solvents, alkaline solutions (10% NaOH), and acidic solutions (10% HCl and 20% H₂SO₄), the qualities that were examined included gloss, impact resistance, hardness, adhesion, and flexibility^33^.

**Table 2:** Mechanical and chemical properties of coating formulations that include derivatives of coumarin 3, 6.

Formulation	Gloss at 60 °C	Impact resistance	Hardness (Kg)	Adhesion	Flexibility	Water resistance	Alkali resistance	Solvent resistance	Acid resistance
Blank sample	9	Cracking	< 2	5B	Pass	Ex	F	Ex	G
Blank & Compound 3	13	Pass	> 2	5B	Pass	Ex	F	Ex	G
Blank & Compound 6	16	Pass	> 2	5B	Pass	Ex	F	Ex	G

The pigment volume concentration (PVC) of the paint was responsible for the gloss level of the coated panels, which stayed within the matte range (15–20 GU) despite the addition of coumarin derivatives 3 and 6 to the coating formulation. Because of the additives’ significant interaction with the steel substrate, the *hardness values*, which varied from 2 kg to more than 2 kg, showed an improvement in surface resistance. This improvement is probably the result of the synthesized compounds’ high electron density, which increases surface stability and cross-linking.

According to the *adhesion* data, all coated films showed good adherence; no flaking was seen at the cut edges, indicating uniform film production and strong interfacial bonding. This could be because the coating matrix and the coumarin derivatives had favorable polar interactions. The *flexibility (bend) test* showed no obvious breaking or detachment, indicating that compounds 3 and 6 did not reduce the flexibility of the film. *Impact resistance* was also enhanced, especially in the compound 6 formulation, which performed better under mechanical stress. The coatings showed outstanding chemical resistance against solvents and aqueous media, which qualifies them for wet situations with low chemical exposure. Although *alkali resistance* was restricted, acid resistance was adequate, indicating that more formulation optimization is required to improve performance in extremely alkaline environments.

### Salt spray test

**Fig. 4:**
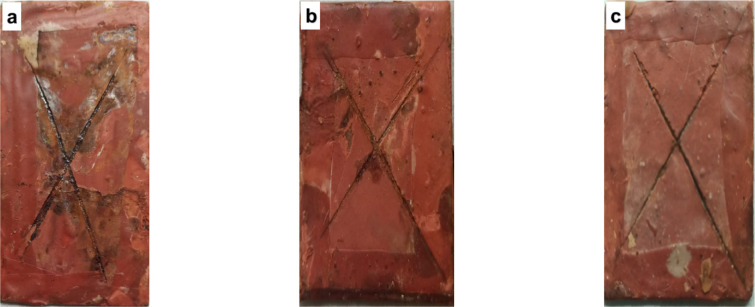
Surface morphology and salt spray corrosion resistance of the coated steel panels after exposure to a 5wt % NaCl solution for 720 h: **(a)** Blank sample, **(b)** Coating formulated with coumarin derivative 3, and **(c)** Coating formulated with coumarin derivative 6.

Lastly, a salt spray test was performed on coated panels to evaluate the impact of coumarin derivatives 3 and 6 on the primer’s protective properties. This is why, as Table 3; Fig. 4 demonstrate, photographic reference standards were used to determine the extent of failure and the percentage of the area that had rusted after being exposed to salt spray fog. Gravity causes salt to accumulate at the bottoms of the samples, where it is noticeably thicker than on the top sides, as seen in Fig. 4a–c.

Table 3; Fig. 4a–c show salt spray resistance data showing that primer formulated with compound **3** or **6** coatings (Fig. 4b and c) greatly enhanced panels’ corrosion resistance compared to blank one (Fig. 4a).

**Table 3:** Salt spray resistance of coated panel.

Panel	Disbanded Area %	Rating Number (ASTM D-1654)
Blank sample	18 ± 0.2	5
Blank & Compound 3	5 ± 0.08	8
Blank & Compound 6	2 ± 0.04	9

According to salt spray testing (Fig. 4), the corrosion inhibition of the primer coumarin derivative 6 formulated coat has superior performance than that of coumarin derivatives 3. The coating’s properties, in contrast to those of bare metal, have a significant impact on how resistant the coated metal is to corrosion. Coumarin-based coat increases corrosion resistance and provides a safer alternative to traditional, hazardous inhibitors like chromates. The primer coumarin formulated coat shows exceptional water and chemical resistance, as well as low toxicity and environmental durability. Coumarin derivatives usually act as fillers that improve the barrier qualities of the primer layer by decreasing the porosity and thus increasing the corrosion resistance. Furthermore, the intertwining of chromene derivatives with primer due to the addition of π-electrons and heteroatoms from phenyl rings may creating a thick network that successfully blocks the entry of oxygen, water, and other corrosive chemicals. Through coordinate bonding, which involves interactions between the electron pairs of heteroatoms, the π-electrons of aromatic rings, and the d-orbitals of the metal surface, coumarin derivatives **3** and **6** chemically adsorb onto the metal surface. The primer formulated with coumarin derivatives **6** coat demonstrated better corrosion resistance than derivatives **3** coat due to the greater quantity of heteroatoms and π-electrons in coumarin derivatives 6 as opposed to derivative **3**.

### Molecular reactivity

The HOMO and LUMO quantum chemical parameters were assessed and presented in Table 4. Ionization energy (I) was determined using HOMO energy, which represents the energy needed to remove an electron from HOMO, as shown below^35–37^.

$${\text{I }} = {\text{ }} - {\text{ E}}_{{{\mathrm{HOMO}}}}$$

Electron affinity (A) is described using the LUMO energy level since the electron occupies the lower energy free orbital in the fundamental type.

$${\text{A }} = {\text{ }} - {\mathrm{E}}_{{{\mathrm{LUMO}}}}$$

Energy gap (ΔE), hardness (η), and softness (σ) are valuable metrics for characterizing chemical reactivity. Associated with the efficiency of corrosion prevention, a reduced energy gap typically signifies increased reactivity and a stronger tendency towards electron transfer reactions, which improve the inhibitor’s efficiency for a molecule to engage with the metal surface, creating a protective layer and preventing corrosion. Moreover, in contrast to the hard molecule, the soft molecule shows superior inhibitory effectiveness. The energy gap (ΔE), chemical hardness (η), and softness (σ) can be computed with the following formulas,

$$\Delta {\text{E }} = {\text{ E}}_{{{\mathrm{LUMO}}}} - {\text{ E}}_{{{\mathrm{HOMO}}}}$$

$$\eta {\text{ }} = {\text{ I }} - {\text{ A}}/{\mathrm{2}}$$

$$\sigma {\text{ }} = {\text{ 1}}/\eta .$$

Table 4 clearly shows that coumarin derivative **6** outperforms coumarin derivative **3** in terms of energy gap (ΔE), hardness (η), and softness (σ) when it comes to inhibiting base metal corrosion. The findings from salt spraying and electrochemical studies aligned with the quantum chemical calculations.

**Table 4:** Quantum computational parameters based on DFT at coumarin derivatives 3 and 6.

Inhibitor	E_HOMO_ (eV)	E_LUMO_ (eV)	ΔE (eV)	I (eV)	A (eV)	η (eV)	σ (eV^− 1^)
Coumarin derivative 3	−7.595	−4.601	2.994	7.595	4.601	1.497	0.668
Coumarin derivative 6	−6.240	−4.325	1.915	6.240	4.325	0.958	1.044

The smaller energy gap of compound 6 (1.915 eV) in contrast to compound 3 (2.994 eV) signifies increased reactivity, implying a stronger tendency to donate electrons at the metal interface, which corresponds with its improved corrosion inhibition.

**Fig. 5:**
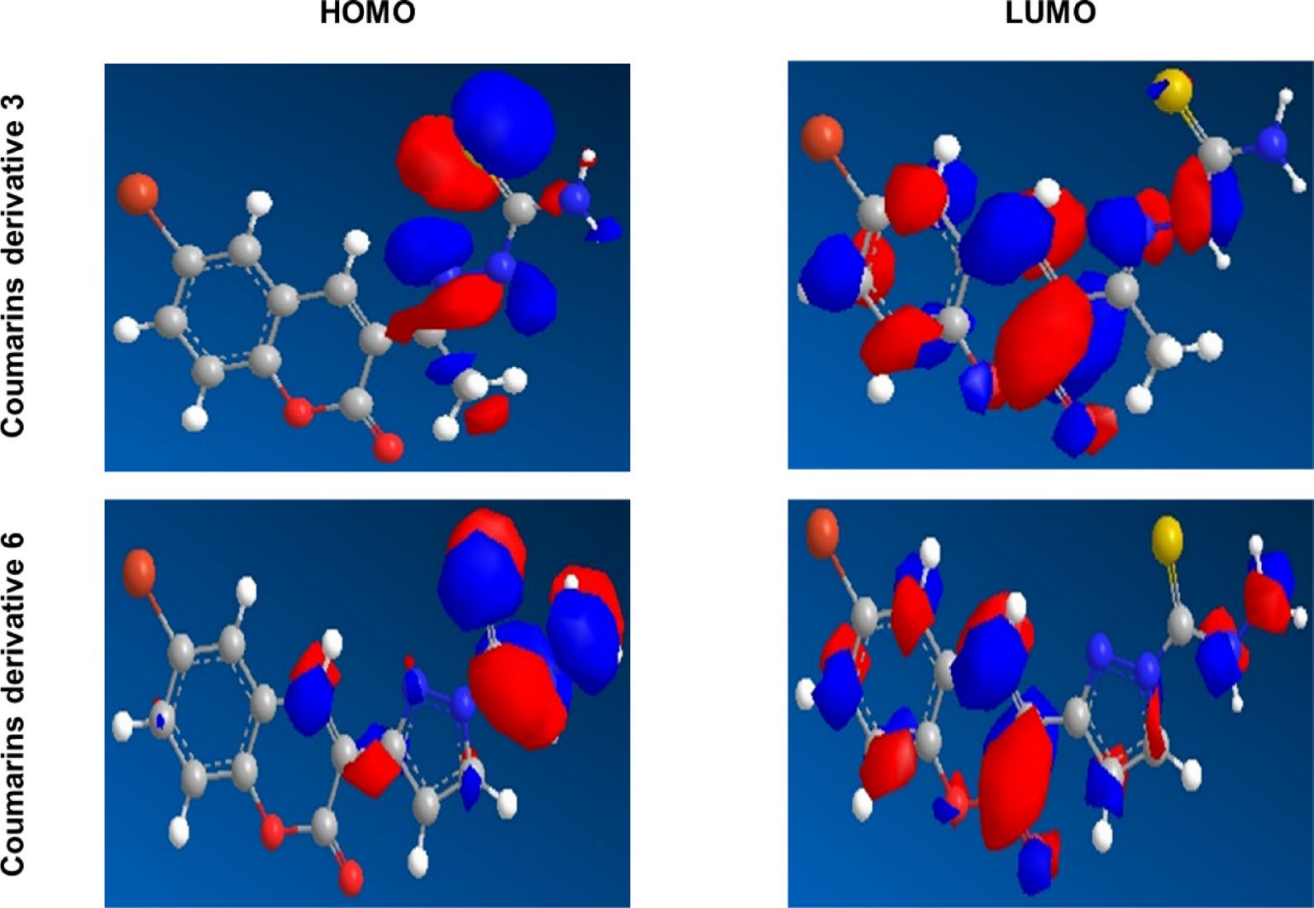
DFT-calculated frontier molecular orbital (FMO) density distributions (HOMO and LUMO) and optimized geometries for the investigated coumarin derivatives 3 and 6.

The spatial arrangement of HOMO and LUMO orbitals for both derivatives, illustrated in Fig. 5, confirms compound 6’s enhanced corrosion inhibition capability by demonstrating its greater electron-donating ability.

## Conclusion

In this study, an environmentally benign, and energy-efficient green protocol was successfully established for the synthesis of two coumarin derivatives utilizing a solvent-free mechanochemical grinding technique. This protocol offers notable benefits over traditional ones, such as high yield, quick reaction time, and elimination hazardous fluid waste. Commercial primer formulations containing these chemicals were used to cover mild steel substrates with protective coatings. The formulated coatings demonstrated excellent physical, mechanical, and chemical qualities, marked by significantly enhanced film hardness, robust cross-hatch adhesion, superb flexibility, and high impact resistance, all achieved without sacrificing gloss or physical film integrity. Furthermore, the cured films exhibited excellent resistance to acids, water, and organic solvents, while alkali resistance was still a problem. According to these results, coumarin derivatives have the potential to be environmentally safe and efficient additives for high-performance anticorrosive coatings, especially in industrial or maritime settings. Overall, the results demonstrate how useful these green-synthesised materials are for developing environmentally friendly corrosion protection systems. These findings confirm the dual role of coumarin derivatives especially compound 6 in enhancing both thermal and anti-corrosion performance. Future studies will focus on extending the electrochemical evaluation (e.g., EIS, Tafel analysis) and surface morphology characterization (SEM, AFM) to provide deeper insight into the protective mechanisms involved.

## Supplementary Information

Below is the link to the electronic supplementary material.

Supplementary Material 1

## Data Availability

The datasets generated and/or analysed during the current study are not publicly available due [reason why data are not public] but are available from the corresponding author on reasonable request.
