# Supplementary material for: Solvent-free synthesis of coumarin derivatives for improved anti-corrosion and mechanical performance of primer coatings
Source: Sci Rep. 2026 Jun 16;16:18708. doi: 10.1038/s41598-026-55719-y (PMC13272963; doi:10.1038/s41598-026-55719-y)
Supplement: Supplementary file 1 — Supplementary Material 1 [file 41598_2026_55719_MOESM1_ESM.docx]

**
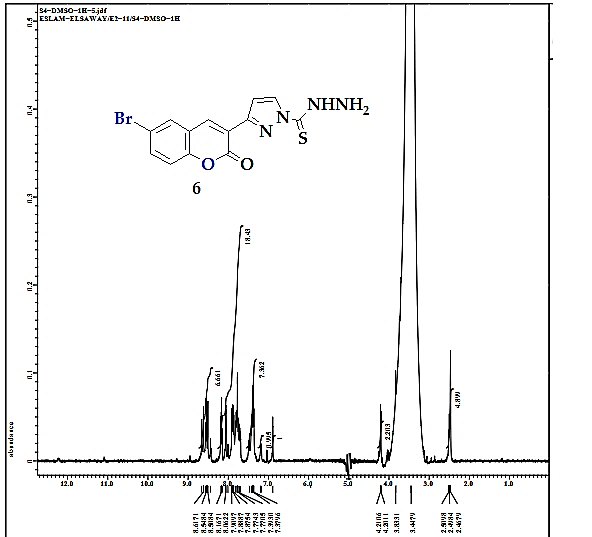
**

**Figure s1. ^1^H NMR spectrum of compound 6.**

**
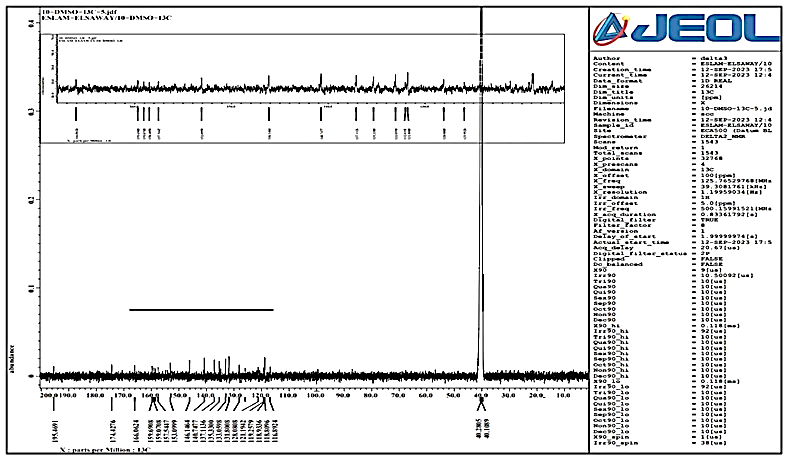
**

**Figure s2. ^13^C NMR of compound 6.**

**
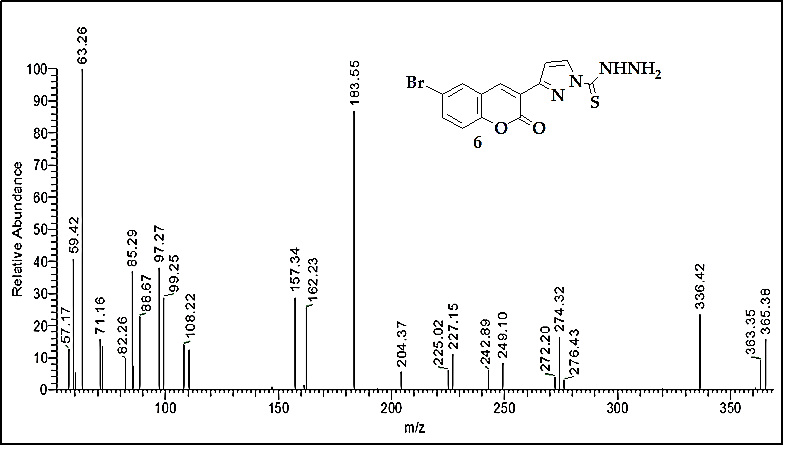
**

**Figure s3. Mass spectrum of compound 6.**


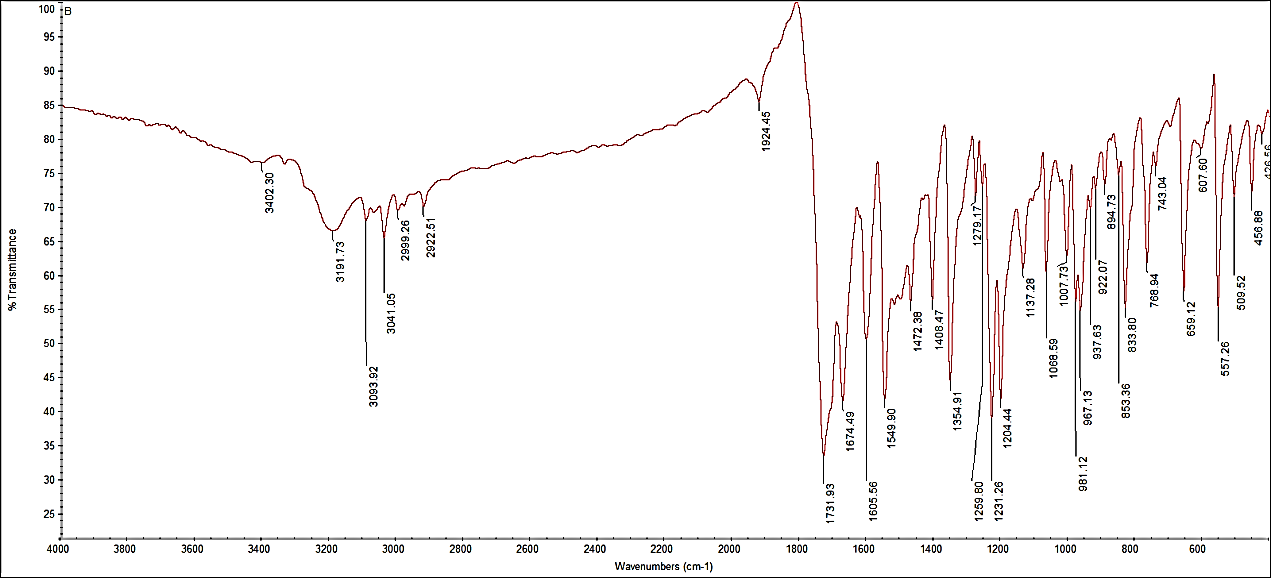


**Figure s4. IR Spectrum for compound 6**
